# Management of ureteral calculi and medical expulsive therapy in emergency departments

**DOI:** 10.4103/0974-2700.76840

**Published:** 2011

**Authors:** Stefano C M Picozzi, Carlo Marenghi, Stefano Casellato, Cristian Ricci, Maddalena Gaeta, Luca Carmignani

**Affiliations:** Department of Urology, Italy; 1 Biometry and Clinical Epidemiology Unit, IRCCS Policlinico San Donato, University of Milan, San Donato Milanese, Milan, Italy

**Keywords:** Emergency department, medical expulsive therapy, nephrolithiasis, renal colic, ureteral stone, ureteral calculi

## Abstract

**Introduction:**

Ureteral stones are a common problem in daily emergency department practice. Patients may be offered medical expulsive therapy (MET1) to facilitate stone expulsion and this should be offered as a treatment for patients with distal ureteral calculi, who are amenable to waiting management. Emergency department clinicians and family practitioners are often in the front line regarding the diagnosis and treatment of symptomatic nephrolithiasis and this commentary is dedicated to them because their decisions directly influence the outcome of the acute stone episode and appropriate referral patterns.

**Materials and Methods:**

The aim of this systematic review and meta-analysis was to understand the role of MET in the treatment of obstructing ureteral calculi. A bibliographic search covering the period from January 1980 to March 2010 was conducted in PubMed, MEDLINE and EMBASE. The searches were restricted to publications in English. This analysis is based on the 21 studies that fulfilled the predefined inclusion criteria.

**Results:**

A metaregression analysis of expulsion time showed a statistically significant advantage in the experimental group, in which the mean expulsion time was 6.2 days compared to 10.3 days in controls. The treatment effect on expulsion rate (P = 0.53) was partially lost as the size of the stones decreased because of the high spontaneous expulsion rate of small stones and the expulsion time was not influenced by pharmacological treatment (*P* = 0.76) if the stone size was smaller than 5 mm. Analysis of the tamsulosin database. A total of 1283 participants were included in the 17 studies. These studies showed that compared to standard therapy or placebo, tamsulosin had significant benefits, being associated with both a higher stone expulsion rate (*P* < 0.001) and reduction of the expulsion time (*P* = 0.02). Reductions in the need for analgesic therapy, hospitalization and surgery are also shown. Analysis of the nifedipine database. The number of participants in each trial ranged from 25 to 70. Compared to standard therapy, the use of nifedipine significantly improved the spontaneous stone expulsion rate (*P* < 0.001). The mean expulsion time was slightly, but not statistically significantly, different (*P* = 0.19) between the treatment and control groups. A possible benefit of nifedipine, in terms of significantly reducing the doses of analgesics required, was reported in three studies. There was no difference between the tamsulosin- and nifedipine-treated groups with regard to expulsion time (*P* = 0.17) or expulsion rate (*P* = 0.79).

**Conclusions:**

Despite all its advantages, MET is rarely used, representing a failure of the translation of medical science into practice. These data raise concerns not only about the quality of care of patients who could benefit from resolution of stones without anaesthetic and surgical risks but also with regard to potential cost savings. MET should be offered as a treatment for patients with distal ureteral calculi who are amenable to a waiting management.

## INTRODUCTION

Ureteral stones are a common problem in daily emergency department practice. In the last 20 years, options for the management of this problem have changed radically. Medical expulsive therapy (MET) has become routine in the treatment of obstructive ureteral calculi, and there is a large body of published data showing the efficacy of such therapy in increasing the expulsion rate and decreasing the expulsion time of stones, thereby reducing lost workdays, urological visits and stone removal procedures,[1–22] even though this treatment did not substantially improve the studied outcomes in two recent trials.[23,24]

Emergency department clinicians and family practitioners are often in the first line in diagnosing and treating symptomatic nephrolithiasis and this systematic review is directed to them because their decisions directly influence the outcome of the acute stone episode and appropriate referral patterns. This article also sheds further light on the issue of MET, with a meta-analysis of the international literature.

### Renal colic and nephrolithiasis

Renal colic caused by nephrolithiasis is common in urological and emergency clinical practice. Urinary stone disease has substantial economic consequences and is of great public health importance, given that the lifetime risk of urolithiasis is estimated to be between 5 and 12% in Europe and in the United States, and that about 50% of patients will have a recurrence of renal colic within 5 years of their first episode.[1,25]

Faced with a new diagnosis of a ureteral stone with a diameter less than 10 mm, in the absence of indications for immediate intervention (such as uncontrolled pain, inadequate renal function, clinical evidence of sepsis or perinephric urine extravasation), an initial treatment option is observation with periodic evaluations.[2] Patients may be offered medical therapy to facilitate stone expulsion during the observation period; in fact, there is considerable evidence that the so-called MET may facilitate and accelerate spontaneous passage of ureteral stones and lower analgesia requirements.[1–22]

### Medical expulsive therapy

The main factors associated with calculus retention include ureteral muscle spasm, submucosal edema, pain and infection within the ureter, and conservative therapy should act on these factors.[4] Since the pioneering work of Borghi *et al*.,[5] in which nifedipine and methylprednisolone were shown to increase the rate of spontaneous stone passage, various aspects of MET have been studied.

Ureteral stones induce ureteral spasm and this is thought to arrest passage of the stone; the corollary of this is that relaxing the ureter in the region of the impacted stone may facilitate passage of the ureteral calculus. Calcium-channel blockers, by modifying the effect of calcium on smooth muscle cells of the ureter, have been proposed to decrease ureteral contractions and, subsequently, the pain of ureteral colic.[5,26] The ureter contains both alpha- and beta-adrenergic receptors. Antagonists of the alpha-1-adrenergic receptor, in particular, inhibit basal tone and decrease peristaltic frequency and amplitude with the consequences of increased fluid transport and decreased intra-ureteral pressure; they also block the conduction of visceral referred pain to the central nervous system, acting on C-fibres or sympathetic postganglionic neurons.[11,27–29]

The presence of a stone in the ureter triggers an inflammatory reaction of the mucosa, which causes various degrees of edema. The most frequently used anti-inflammatory drugs in this context are corticosteroids, which are given in association with alpha-1-adrenergic receptor antagonists and calcium-channel blockers because of their action of decreasing edema and inflammation and, thereby, relieving an obstacle to the passage of the stone.[26,30–32] Corticosteroids should, however, only be used for short periods in order to avoid the many adverse effects associated with prolonged therapy.[33] The role of corticosteroid therapy alone has not been investigated.[26,30]

Antibiotics and analgesic therapy complete the treatment regimen.

### Evidence-based data

Evience-based medicine aims to apply the best available evidence gained from the scientific method to medical decision-making. It seeks to assess the quality of evidence of the risks and benefits of treatments. MET can have side effects and every patient should be counseled on the benefits and the risks of the drugs used and should be informed that they are administered for an “off-label” use.

## MATERIALS AND METHODS

The aim of this systematic review and meta-analysis was to understand the role of MET in the treatment of obstructing ureteral calculi. Clinical outcomes of interest were spontaneous stone expulsion rate and mean time of expulsion. We also qualitatively evaluated: (i) control of colic pain, determined by the number of colic episodes and analgesic requirements; (ii) reduction of hospitalization, determined by the number of hospital admissions and surgical interventions; and (iii) adverse effects, determined by the number of patients who discontinued MET because of side effects related to the drugs used.

### Search strategy

Studies were identified by searching electronic databases and scanning reference lists of articles. A bibliographic search covering the period from January 1980 to March 2010 was conducted in PubMed, MEDLINE and EMBASE. Additional hand searches of the reference lists of included studies, reviews, meta-analyses and guidelines on the use of MET for ureteral stones were performed. The searches were restricted to publications in English.

### Study selection

The most commonly used and investigated agents in MET are tamsulosin and nifedipine. Identified studies were reviewed and selected if they reported the use of either of these two drugs in MET. Inclusion or exclusion of studies was performed hierarchically based first on the title of the report, then on the abstract, and finally on the contents of the full text. A study was accepted for inclusion on the basis of agreement of two investigators (SCMP and CM); any disagreement on study inclusion was resolved by consulting a third investigator (LC).

Database searches yielded 86 references. Exclusion of irrelevant references left 24 references describing studies. We excluded three further references because they were not in English. This analysis is based on the 21 studies that fulfilled the predefined inclusion criteria.

### Study classification

Studies were classified according to the Cochrane Intervention Meta-analysis Handbook into non-randomized comparative studies (including non-randomized, controlled trials, retrospective cohort studies and historically controlled trials) and in randomized clinical trials.

### Data extraction and assessment of quality

One author (SCMP) extracted the following data from included studies and entered them into the data extraction form. A second author (CM) checked the extracted data to ensure data quality. Disagreements were resolved by discussion between the two review authors; if no agreement could be reached, it was planned that a third author would decide (LC). The quality of studies was scored using the methods of the US Preventive Services Task Force.[34,35]

The US Preventive Services Task Force classifies a study as “good” if it evaluates relevant available screening tests, uses a credible reference standard, interprets the reference standard independently of the screening test, shows reliability of the test assessed, has few or handles indeterminate results in a reasonable manner and includes a large number of patients (more than 100 broad-spectrum cases); as “fair” if it evaluates relevant available screening tests, uses reasonable although not best standards, interprets the reference standard independently of the screening test, has a moderate sample size (50–100 subjects) and a “medium” spectrum of patients; and as “poor” if it has a fatal flaw such as using an inappropriate reference standard, administering a screening test improperly, biased ascertainment of a reference standard, and has a very small sample size or very narrow selected spectrum of patients.

### Statistical analysis

An overall quantitative evaluation was made of all the studies included. Both the fixed and the random effect models were used to evaluate the overall effects on expulsion rate.[36] Expulsion time analysis was performed using a weighted meta-regression model using the GLM procedure of the SAS software package; expulsion rate analysis was performed by fixed and random effect models using Rev-Man 5. The degree of heterogeneity among the trials was assessed by the *I*-squared (*I*^2^) statistic. The extent to which study-level variables explained heterogeneity in predicting the outcome was then explored by fitting fixed effects meta-regression models to account for calculus diameter, drug usage and kind of drug.

We analyzed the presence of potential publication and small study bias applying the funnel plot. We integrated the visual inspection of the funnel plot with the test proposed by Harbord.[37]

All analyses were performed using SAS software package version 9.1.3 (SAS Institute Inc., SAS 9.1.3 Help and Documentation, Cary, NC, USA: SAS Institute Inc., 2000–2004) and RevMan 5 (Review Manager, version 5.0; Copenhagen, Denmark: The Nordic Cochrane Centre, The Cochrane Collaboration, 2008).

## RESULTS

### Heterogeneity evaluation among studies

**Figure 1:**
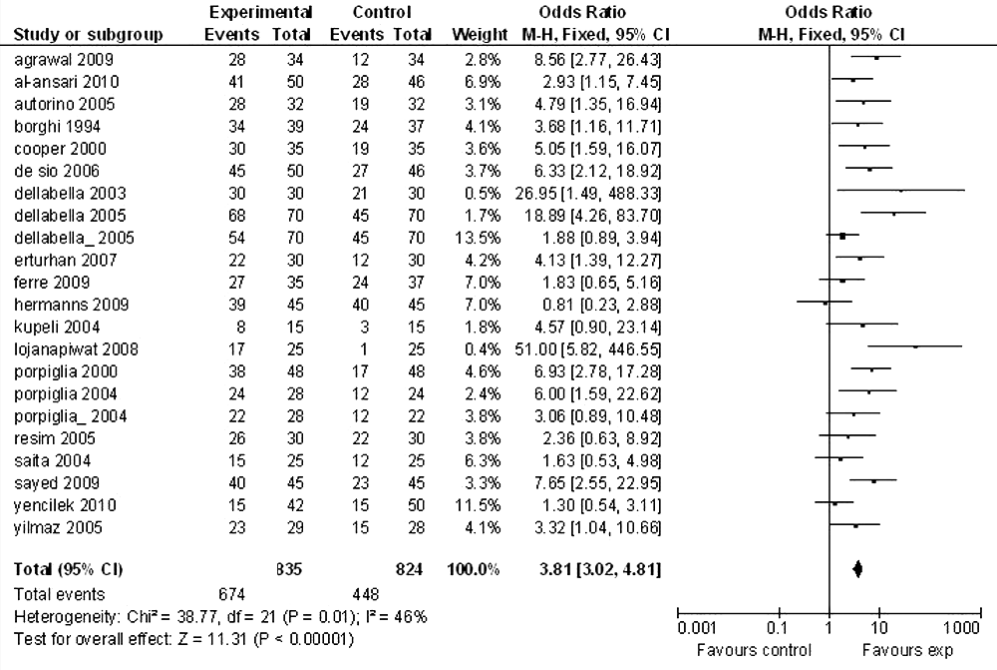
Forest plot of expulsion rate estimate by the fixed effect model. The overall odds ratio estimate 3.81 (3.02;4.81) favors experimental groups showing that the probability of calculus expulsion is about four times higher if either of the two pharmacological approaches (tamsulosin or nifedipine) is applied

All of the included studies belong to the general design of the clinical trials and all studies have a similar sample size; also, the outcomes reported agree that the experimental groups have a higher expulsion rate and a lower expulsion time; moreover, there was not an appreciable difference between results from the random and fixed effect analyses performed. Because of the large homogeneity among studies, the fixed effect analysis was reported [Figure 1].

### Overall analysis of the outcomes

We found an overall significant effect of experimental versus control management in analyses regarding both expulsion rate and expulsion time. The fixed effect model applied to expulsion rate [Figure 1] showed a significant odds ratio for the experimental group [odds ratio estimate = 3.81 (3.02;4.81)], with an *I*^2^ of 46%. A metaregression analysis of expulsion time also showed a statistically significant advantage in the experimental group, in which the mean expulsion time was 6.2 (3.6;8.7) days compared to a mean time of expulsion of 10.3 days (7.8;12.9) in controls.

### Evaluation of stone diameter effect

As previously reported, the treatment effect on expulsion rate (*P* = 0.53) was partially lost as the size of the stones decreased because of the high spontaneous expulsion rate of small stones;[3] the expulsion time was not influenced by pharmacological treatment (*P* = 0.76) if the stone size was smaller than 5 mm.

### Publication bias assessment

**Figure 2:**
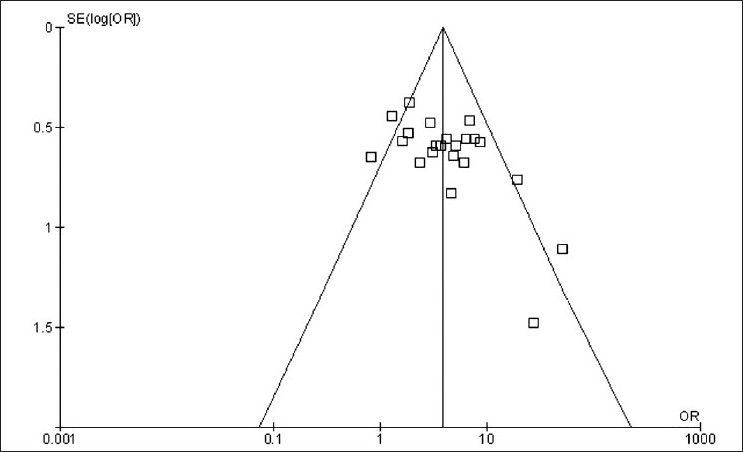
Funnel plot of expulsion rate odds ratio and standard error. The vertical line represents the overall odds ratio estimate, oblique lines define a region within which 95% of points might lie in the absence of both heterogeneity and publication bias

The funnel plot [Figure 2] was slightly asymmetrical suggesting that there might be a publication bias. According to Harbord,[37] the regression analysis applied to the relation between the odds ratio and and the logarithm of its standard error was statistically significant because of the studies by Della Bella and Lojanapiwat.[7,11,15]

### Analysis of the tamsulosin database

Table 1 summarizes the characteristics of all the included studies regarding the use of the selective alpha-1A/1D-adrenoceptor antagonist, tamsulosin. A total of 1283 participants were included in the 17 studies. The number of participants in each trial ranged from 15 to 70. All the studies were published after 2000. Five studies were from Italy, five from Turkey and Egypt, India, Qatar, Thailand, and the United States, the Slovak Republic and Switzerland provided one study each.

**Table 1:** Data regarding the use of tamsulosin 0.4 mg extracted from the studies listed by authors and year of publication

Author	Treatment/ control patients	Stone location	Stone size (mm)	Intervention	Follow up	Mean time to expulsion (days)	Stone expulsion rate (%)	Control of colic pain	Need for hospitalization	Treatment discontinuation
Cervenacov, 2002[6]	51/51	Lower ureter	NR	Tamsulosin/control	1 week	3.1/3.4	80.4/62.8	Reported	NR	None
Dellabella, 2003[7]	30/30	Juxtavesical tract	6.7/5.8	Tamsulosin/l floroglucine-trimetossibenzene	4 weeks	2.74/4.63	100/70	Reported	Decreased	None
Kùpeli, 2004[8]	15/15	Lower ureter	4.7/4.9	Tamsulosin/control	2 weeks	NR	53.3/20	NR	NR	None
Porpiglia, 2004[9]	28/28	Juxtavesical tract	5.4/5.3	Tamsulosin/control plus deflazacort	4 weeks	7.9/12	85/43	Reported	Decreased	One patient
Yilmaz, 2005[10]	29/28	Juxtavesical tract	6/6.07	Tamsulosin/control	2 weeks	6.31/10.54	79.3/53.6	Reported	Decreased	None
Dellabella, 2005[11]	70/70	Lower ureter	7.2/6.2	Tamsulosin/phloroglucinol plus cotrimoxazole and deflazacort	4 weeks	3/5	97/64	Reported	Decreased	None
Resim, 2005[11]	30/30	Lower ureter	7.8/7.8	Tamsulosin/control	6 weeks	NR	86.6/73.3	Reported	Decreased	None
Autorino, 2005[4]	32/32	Distal ureter	6.5/5.7	Tamsulosin/control	2 weeks	4.8/7.4	88/60	Reported	Decreased	None
De Sio, 2006[13]	50/46	Distal ureter	6.9/6.4	Tamsulosin/control	2 weeks	4.4/7.5	90/58.7	Reported	Decreased	None
Erturhan, 2007[14]	30/30	Distal ureter	7.1/6.8	Tamsulosin/control	3 weeks	6.4/12.2	73.3/40	Reported	NR	None
Lojanapiwat, 2008[15]	25/25	Distal ureter	6.3/6.7	Tamsulosin/control	4 weeks	10.7/23	68/4	No difference	Decreased	None
Sayed, 2008[16]	45/45	Distal ureter	6.8/6.4	Tamsulosin/control	4 weeks	7.32/12.53	88.9/51.1	Reported	Decreased	None
Hermanns, 2009[23]	45/45	Distal ureter	4.1/3.8	Tamsulosin/control	3 weeks	7/10	86.7/88.9	No difference	Decreased	One patient
Ferre, 2009[24]	35/37	Distal ureter	3.5/3.8	Tamsulosin/control	2 weeks	1/3	77.1/64.9	No difference	NR	None
Agrawal, 2009[17]	34/34	Distal ureter	6.17/6.35	Tamsulosin/control	4 weeks	12.3/24.5	82.3/35.2	NR	NR	None
Yencilek, 2010[18]	42/50	Distal ureter	6.4/6.6	Tamsulosin/control	4 weeks	8.4/11.6	35.7/30	Reported	Decreased	None
Al-Ansari, 2010[19]	50/46	Distal ureter	5.88/6.04	Tamsulosin/control	4 weeks	6.4/9.8	82/61	Reported	Decreased	None

THE CONTROL OF COLIC PAIN WAS DETERMINED FROM THE REDUCTION IN COLIC EPISODES AND ANALGESIC REQUIREMENTS, NEED FOR HOSPITALIZATION WAS DETERMINED FROM THE REDUCTION OF HOSPITAL ADMISSIONS AND SURGICAL INTERVENTIONS; NR: NOT REPORTED

These studies showed that compared to standard therapy or placebo, tamsulosin had significant benefits, being associated with both a higher stone expulsion rate and reduction of the expulsion time. The expulsion rate was statistically different (*P* < 0.001) between the treatment and control groups with the odds ratio estimate being 3.74 (1.95;7.15). The mean expulsion time was also statistically different (*P* = 0.02) between the treatment and control groups [mean expulsion time in the treatment group = 6.02 (3.50;8.54) days; mean expulsion time in the control group = 10.3 (7.79;12.82) days]. Reductions in the need for analgesic therapy, hospitalization and surgery are also shown in Table 1. Adverse events rarely led to patients withdrawing from MET and were reversible after discontinuation of the drug administered.

Most of the studies used tamsulosin, probably because of its routine use by urologists and excellent tolerability. Limited direct comparative data indicate that other alpha-antagonists (doxazosin and terazosin) may have similar efficacy.

### Analysis of the nifedipine database

Table 2 shows the results of the use of nifedipine in the studies analyzed. A total of 488 participants were included in the six studies considered. The number of participants in each trial ranged from 25 to 70. One study was published in the 1990s, while the other five were published after 2000. Five studies were from Italy and one from the United States. Compared to standard therapy, the use of nifedipine significantly improved the spontaneous stone expulsion rate and slightly reduced the time to stone expulsion. The expulsion rate was statistically different (*P* < 0.001) between the treatment and control groups with an odds ratio estimate of 3.34 (1.86;6.00). The mean expulsion time was slightly, but not statistically significantly, different (*P* = 0.19) between the treatment and control groups [mean expulsion time in the treatment group = 8.06 (3.73;12.38) days; mean expulsion time in the control group = 11.92 (7.58;16.27) days]. A possible benefit of nifedipine, in terms of significantly reducing the doses of analgesics required, was reported in three studies.[9,11,20]

**Table 2:** Data regarding the use of nifedipine 30 mg extracted from the studies listed by authors and year of publication

Author	Treatment/ control patients	Stone location	Stone size (mm)	Intervention	Follow up	Mean time to expulsion (days)	Stone expulsion rate (%)	Control of colic pain	Need for hospitalization	Treatment discontinuation
Borghi, 1994[5]	39/37	Ureter	6.7/6.8	Nifedipine/control plus methylprednisolone	7 weeks	11.2/16.4	87/65	NR	Decreased	Four patients
Porpiglia, 2000[20]	48/48	Lower ureter	5.8/5.5	Nifedipine and deflazacort/ control	4 weeks	7/20	79/35	Reported	Decreased	Two patients
Cooper, 2000[21]	35/35	Ureter	3.86/3.91	Nifedipine and prednisone/ control	7 weeks	12.6/11.5	86/54	NR	Decreased	NR
Saita, 2004[22]	25/25	Ureter	12/12.8	Nifedipine and prednisone/ prednisone	3 weeks	6/10	60/48	NR	NR	Six patients
Porpiglia, 2004[9]	28/28	Juxtavesical tract	4.7/5.3	Nifedipine/control plus deflazacort	4 weeks	9.3/12	80/43	Reported	Decreased	One patient
Dellabella, 2005[11]	70/70	Lower ureter	6.2/6.2	Nifedipine/phloroglucinol plus cotrimoxazole and deflazacort	4 weeks	5/5	77/64	Reported	No difference	None

THE CONTROL OF COLIC PAIN WAS DETERMINED FROM THE REDUCTION IN COLIC EPISODES AND ANALGESIC REQUIREMENTS, NEED FOR HOSPITALIZATION WAS DETERMINED FROM THE REDUCTION OF HOSPITAL ADMISSIONS AND SURGICAL INTERVENTIONS; NR: NOT REPORTED

Adverse effects that caused treatment discontinuation seemed to occur more frequently in patients treated with nifedipine than in the patients treated with tamsulosin.

### Evaluation of treatment efficacy

There was no difference between the tamsulosin- and nifedipine-treated groups with regard to expulsion time (*P* = 0.17) or expulsion rate (*P* = 0.79).

## DISCUSSION

Treatment modalities for ureteral stones have greatly changed during the last 20 years, especially following the introduction of minimally invasive procedures such as extra-corporeal shock wave lithotripsy and ureterorenoscopy. Although these procedures are effective, they are not risk-free and are expensive.

Despite all its advantages, MET is rarely used, representing a failure of the translation of medical science into practice. Hollingsworth *et al*. reported a 1.1% overall prevalence of MET use between 2000 and 2006 in emergency departments in the USA, with a missed opportunity of sparing approximately 260,000 individuals annually from stone surgery.[38] These data raise concerns not only about the quality of care of patients who could benefit from resolution of stones without anaesthetic and surgical risks but also with regard to potential cost savings. For example, in Italy, the estimated cost of surgery for urolithiasis ranges from 1849 Euros for non-complicated ureterorenoscopy to 501 Euros for day-hospital shock-wave lithotripsy, without taking into consideration indirect costs; in contrast, a 30-day course of alpha-blockers costs around 10 Euros.[39–42]

There are various possible explanations for the underuse of MET, but as recently recognized in our daily clinical practice, confirming previous reports in the international literature, the most relevant is the gap between the different clinical figures involved in the management of patients with nephrolithiasis, such as family practitioners, emergency department physicians and urological surgeons who care for symptomatic patients, due to the almost exclusively urological profile of publications and guidelines regarding MET.

As in our institution, this bias could be resolved by the creation of specific, regularly updated guidelines shared by specialists (urologists) and emergency department physicians, developed on the basis of international guidelines, in the context of continuous close collaboration between different sub-specialists and emergency department clinicians.

## CONCLUSIONS

MET should be offered as a treatment for patients with distal ureteral calculi who are amenable to a waiting management. Benefits associated with MET are a shorter time to stone expulsion and less need for analgesic drugs and hospitalization for treatment. MET is cost-effective for the management of distal ureteral stones.
